# The TALE face of Hox proteins in animal evolution

**DOI:** 10.3389/fgene.2015.00267

**Published:** 2015-08-18

**Authors:** Samir Merabet, Brigitte Galliot

**Affiliations:** ^1^Centre National de Recherche Scientifique, Institut de Génomique Fonctionnelle de LyonLyon, France; ^2^Institut de Génomique Fonctionnelle de Lyon, Ecole Normale Supérieure de LyonLyon, France; ^3^Department of Genetics and Evolution, Faculty of Science, Institute of Genetics and Genomics in Geneva, University of GenevaGeneva, Switzerland

**Keywords:** Hox, PBC, Meis, Metazoa, patterning, early-branching phyla, HX, SPIMs

## Abstract

Hox genes are major regulators of embryonic development. One of their most conserved functions is to coordinate the formation of specific body structures along the anterior-posterior (AP) axis in Bilateria. This architectural role was at the basis of several morphological innovations across bilaterian evolution. In this review, we traced the origin of the Hox patterning system by considering the partnership with PBC and Meis proteins. PBC and Meis belong to the TALE-class of homeodomain-containing transcription factors and act as generic cofactors of Hox proteins for AP axis patterning in Bilateria. Recent data indicate that Hox proteins acquired the ability to interact with their TALE partners in the last common ancestor of Bilateria and Cnidaria. These interactions relied initially on a short peptide motif called hexapeptide (HX), which is present in Hox and non-Hox protein families. Remarkably, Hox proteins can also recruit the TALE cofactors by using specific PBC Interaction Motifs (SPIMs). We describe how a functional Hox/TALE patterning system emerged in eumetazoans through the acquisition of SPIMs. We anticipate that interaction flexibility could be found in other patterning systems, being at the heart of the astonishing morphological diversity observed in the animal kingdom.

## Introduction

The phenotypic diversity observed in the animal kingdom arose from genetic innovations that modulate developmental processes, a step in evolution that often precedes speciation events (Gould, 1992; Arthur, 2002). A major challenge in biology is to characterize these genetic innovations and to understand how they impact developmental processes. Remarkably, the specification of body plans and body parts in species as different as humans or flies is controlled by a relatively small and highly conserved genetic repertoire called the “genetic toolkit” (True and Carroll, 2002; Erwin, 2009). This genetic toolkit, which acts at restricted stages of embryonic development, encodes for molecules involved in cell-cell communication, and gene regulation (Mann and Carroll, 2002). Components of the genetic toolkit are described in several bilaterian species to form character identification networks (Wagner, 2007), or kernels (Davidson and Erwin, 2006), which are part of large developmental networks that underlie body plan development (Davidson and Erwin, 2006). Several members of the genetic toolkit are also expressed in choanoflagellates, indicating that they originated prior to the emergence of the first metazoans (King et al., 2003; King, 2004; Wenger and Galliot, 2013).

**Figure 1:**
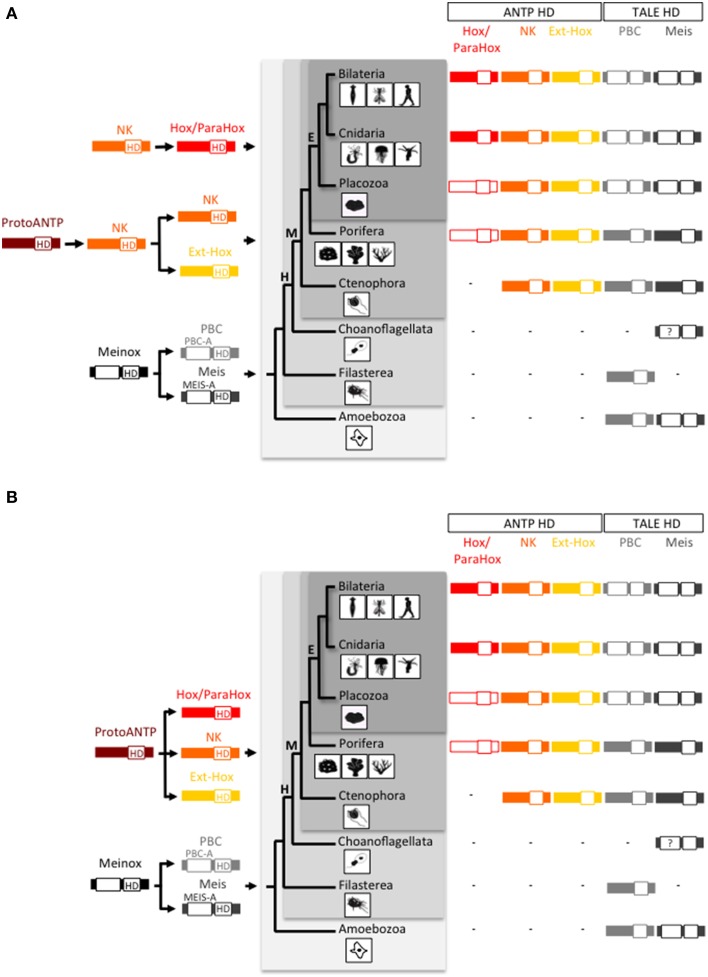
**Origin and early evolution of ANTP- and TALE-class gene families. (A)** First evolutionary scenario whereby the Hox/ParaHox family would have derived from a NK member in the Eumetazoan ancestor. **(B)** Second evolutionary scenario, also named the “ghost loci hypothesis,” whereby the main homeobox gene families (Hox/ParaHox, NK, and Ext-Hox) would have derived from a ProtoANTP cluster of homeobox genes already present in the Last Common Ancestor (LCA) of metazoans. The recent finding of a ParaHox-like gene in Porifera (Fortunato et al., 2014) actually supports the second scenario. Note that only one ParaHox member (symbolized by the absence of red filling) is found in Placozoa [annotated as a Gsx-like: (Schierwater et al., 2008a)] and Porifera [annotated as a Cdx-like: (Fortunato et al., 2014)] and that no Hox or ParaHox gene has been annotated in Ctenophora so far. In comparison, the PBC and Meis families originated earlier in the life tree, with representatives already present in unicellular phyla (Amoebozoa and Filasterea). Graded gray backgrounds highlight Eumetazoa (E), Metazoa (M), Holozoa (H), and Unikonta (U) super phyla. The homeodomain (HD) is indicated in each protein. PBC-A and Meis-A are domains required for the PBC/Meis partnership. Question mark in Choanoflagellata is for incomplete protein sequence of Meis. Animal drawings were taken from Ryan and Baxevanis (2007).

The large majority of contemporary animals belong to Bilateria, which are characterized by three embryonic germ layers (ectoderm, mesoderm, endoderm) and a bilateral symmetry that results from the orthogonal intersection of two longitudinal axes, the anterior-posterior (AP) axis (also referred to as the primary axis), and the dorso-ventral (DV) axis (also referred to as the secondary axis). Bilaterians radiated during the Cambrian period some 500–550 million years ago. Other extant non-bilaterian species belong to Porifera (sponges), Ctenophora, Placozoa (Trichoplax), and Cnidaria, whose ancestors predate the Cambrian explosion, thus often named early-branched phyla (Figure 1). With the exception of Placozoa, species from these early-branched phyla display different types of symmetry, either radial (as seen in sponge larvae, some adult sponges, and in most cnidarians), or biradial (as seen in ctenophores), or partly bilateral (as seen in sea anemone species that belong to the anthozoan class of cnidarians). These various symmetries are especially evident during embryogenesis and larval stages and depend on the formation of a primary body axis (Ryan and Baxevanis, 2007).

Cnidaria, a sister group to Bilateria, share with them typical features of eumetazoans, i.e., an ectodermal layer that differentiates as an epidermis, an endodermal layer that differentiates as a gut, and a nervous system, which, at the oral pole/extremity, allows an active feeding behavior. Also, Cnidaria includes a large variety of taxa with a wide spectrum of morphological diversity. All together, these characteristics place Cnidaria at a key phylogenetic position for tracing the emergence of molecular innovations that underlie developmental changes and diversification in animal evolution (Steele et al., 2011). Representative(s) of the main gene families involved in the specification of eumetazoan features are also found in Cnidaria (Martindale, 2005). Their study is however more challenging, due to the lack of advanced genetic tools that could allow establishing transgenic animals for stable gene expression or extinction in a tissue- and/or stage-specific manner.

Among the different conserved developmental gene families are the Hox genes, which are considered as the “Rosetta Stone” of the genetic toolkit. Hox genes were initially discovered in *Drosophila*, then rapidly investigated in vertebrate species, showing striking conserved features throughout bilaterian lineages (Lewis, 1978; McGinnis and Krumlauf, 1992; Kmita and Duboule, 2003). These conserved properties have been discussed in several reviews and relate to their clustered genomic organization that constrains embryonic expression (Duboule, 2007), but also to the presence of several typical protein signatures (Ogishima and Tanaka, 2007; Merabet et al., 2009). Modifications in Hox gene expression or in Hox protein function have been linked to several morphological innovations during the evolution of bilaterians (Pearson et al., 2005; Heffer et al., 2013). The presence of Hox genes in Cnidaria therefore raised the question of their role in the emergence of innovations shared by cnidarians and bilaterians, as well as in the emergence of innovations responsible for the morphological diversity observed among cnidarian species.

The most spectacular observation came from the embryo of the cnidarian sea anemone *Nematostella vectensis*, where several Hox-related genes show a staggered-like expression pattern along the oral-aboral (OA) axis. This expression profile led to the proposition that the cnidarian OA axis could be homologous to the bilaterian AP axis (Finnerty et al., 2004; Matus et al., 2006). The OA expression profile of *Nematostella* Hox genes is however neither conserved in other cnidarian lineages nor strictly following the collinear rules normally observed in Bilateria (Gauchat et al., 2000; Finnerty et al., 2004; Kamm et al., 2006; Ryan et al., 2007; Chiori et al., 2009). These additional observations led to the opposite conclusion that a Hox patterning system is likely not existing in Cnidaria (Kamm et al., 2006).

Surprisingly, the question of the evolution of Hox patterning mechanisms is rarely approached at the protein level, in particular by considering members of the PBC and Meis families. PBC and Meis are crucial patterning cofactors of Hox proteins along the AP axis, a partnership that is evolutionarily-conserved throughout Bilateria (Moens and Selleri, 2006; Mann et al., 2009). PBC and Meis belong to the TALE (Three Amino acids Loop Extension) class of homeodomain (HD)-containing transcription factors (Bürglin, 1997), are widely conserved across metazoans and can therefore be used as a molecular hallmark of the Hox patterning system. In this review, we report how the intricate interaction properties between Hox and TALE proteins were progressively acquired in pre-bilaterian animal evolution to eventually constitute a major patterning system.

### Origin and early evolution of the Hox/ParaHox and PBC/Meis gene families

Hox proteins belong to the ANTP (Antennapedia) class of HD-containing transcription factors. This class contains two large groups of sister gene families: (i) the non-Hox ANTP-class group, which includes the NK and Extended (Ext)-Hox gene families, and (ii) the Hox/ParaHox genes (Garcia-Fernàndez, 2005). The Hox gene family is usually found organized in clusters and contains several paralog groups (PGs) that are themselves classified into anterior (PG1-3), central (PG4-8), and posterior (PG9-14) (Duboule, 2007). The ParaHox family contains three clustered genes initially discovered in the cephalochordate amphioxus (Brooke et al., 1998), and named Gsx, Pdx/Xlox, and Cdx. ParaHox genes share common ancestors with specific Hox gene families, Gsx, and Pdx/Xlox with the anterior PG2/PG3, Cdx with the posterior PG9 (Quiquand et al., 2009).

Two different scenarios are proposed to explain the evolutionary history of the Hox/ParaHox gene family with regard to the other ANTP-class members. In the first one, the Hox/ParaHox family is specific to eumetazoans (which regroup Bilateria, Cnidaria, and Placozoa) and would have originated from duplications of a ProtoHox gene derived from NK genes and related to Evx/Mox (Ext-Hox family) (Gauchat et al., 2000; Minguillón and Garcia-Fernàndez, 2003; Larroux et al., 2007; Quiquand et al., 2009; Ryan et al., 2010) (Figure 1A). This scenario is supported by the presence of a Gsx ParaHox gene in the genome of the placozoan *Trichoplax adhaerens* (Schierwater and Kuhn, 1998; Schierwater et al., 2008b), the presence of several NK representatives and the absence of Hox/ParaHox genes in the genome of the ctenophores *Mnemiopsis leidyi* (Ryan et al., 2010) and *Pleurobrachia bachei* (Moroz et al., 2014) and the demosponge *Amphimedon queenslandica* (Srivastava et al., 2010). However, this scenario is challenged by the “ghost loci hypothesis”, which postulates that Hox/ParaHox genes were already present in the Last Common Ancestor (LCA) of metazoans and secondarily lost in Porifera over evolutionary times (Mendivil Ramos et al., 2012). In this second scenario, the Hox/ParaHox and NK families emerged independently from a common ProtoANTP ancestor gene (Figure 1B). The recent finding of a Cdx-like ParaHox gene in the genome of two calcareous sponges (Fortunato et al., 2014), now argues in favor of the ghost loci hypothesis.

In addition to ANTP, other classes of HD-containing transcription factors are also present in early branch phyla. These include the Paired-like, Pax, Pou, Lim, Six, and TALE classes (Galliot and de Vargas, 1999; Larroux et al., 2008; Srivastava et al., 2010; Holland, 2013; Fortunato et al., 2014). Members of the TALE class contain an atypical 63-residues long HD, due to the presence of three extra residues in between the helices 1 and 2 of the HD (Mukherjee and Bürglin, 2007). TALE class members are among the most ancient transcription factors in eukaryotes, with several of them present in unicellular organisms, plants and fungi (Bürglin, 1997, 1998), therefore predating the origin of animals. Interestingly, TALE-class members can interact with different types of HD-containing proteins in plants (Bellaoui et al., 2001; Hackbusch et al., 2005; Kanrar et al., 2006; Hay and Tsiantis, 2010), fungi (Keleher et al., 1989; Stark and Johnson, 1994; Carr et al., 2004), and animals (Bürglin, 1998).

The TALE class comprises five families (PBC, Meis, Iro, TGIF, and MKX), among which two, PBC and Meis are known to interact with ANTP members (Mukherjee and Bürglin, 2007). PBC and Meis were already present before multicellular organisms appeared (King et al., 2008; Clarke et al., 2013; Suga et al., 2013) and (Figure 1). Animal representatives of PBC and Meis include the Pbx1-4 or Extradenticle (Exd) and Meis1-3 or Homothorax (Hth) proteins, as named in mammals and in the fruit fly *Drosophila melanogaster*, respectively. PBC and Meis families originated from the duplication of a common ancestor gene named MEINOX, and this duplication was proposed to coincide with the apparition of the first Hox cluster in metazoans (Bürglin, 1998). Genome comparisons between early-branched metazoan species and unicellular organisms now establish that the PBC/Meis duplication predated the ANTP class and therefore the Hox/ParaHox family. Thomas Bürglin was however the first one to consider the partnership between Hox and TALE proteins as an informative molecular hallmark to trace the origin of the Hox patterning system in Metazoa (Bürglin, 1998).

### The ground state of Hox/TALE interaction networks in Bilateria: role of the hexapeptide (HX) motif

**Figure 2:**
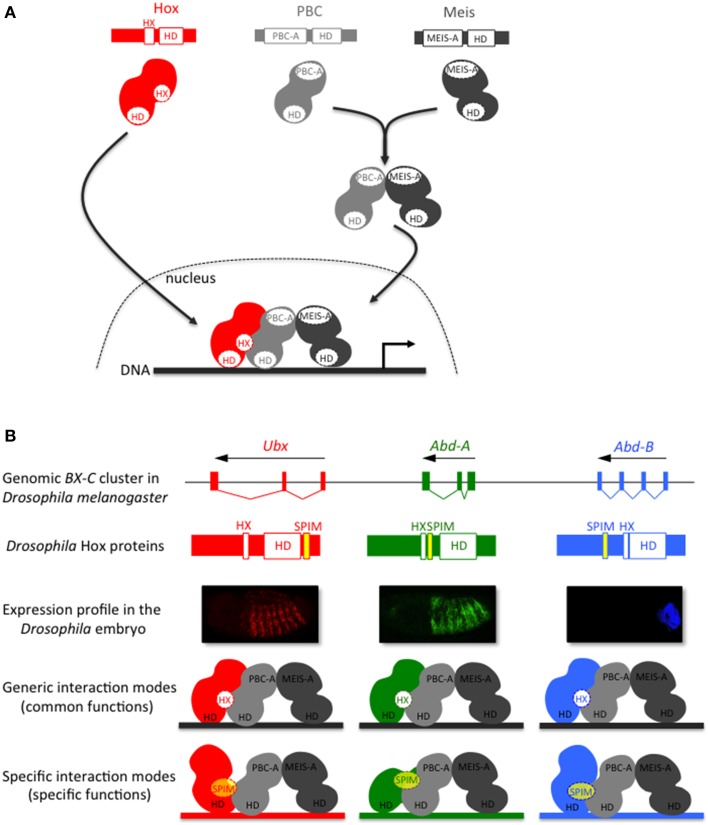
**The Hox-TALE interaction network: role of generic (HX motif) and specific PBC interaction motifs (SPIMs). (A)** Generic association mode between Hox and TALE proteins. The interaction between Meis and PBC allows the nuclear translocation of PBC. The hexapeptide (HX) motif, present in Hox proteins of all bilaterian lineages, is necessary and sufficient for the generic association mode of the Hox/TALE complex on DNA. **(B)** Model for the role of SPIMs in specifying patterning functions among *Drosophila* Hox proteins. The usage of SPIMs allows each Hox protein of the Bithorax complex (BX-C) to adopt different conformation modes with the TALE cofactors and regulate different target genes *in vivo* (as illustrated by the color code). The placement of SPIMs (highlighted in yellow) in Ultrabithorax (Ubx) and Abdominal-A (Abd-A) reflects the position of the UbdA and TDWM motifs, respectively (Hudry et al., 2012). The placement of the SPIM in Abdominal-B (Abd-B) is speculative.

The formation of Hox/PBC/Meis complexes in Bilateria is described to rely on Hox-PBC and PBC-Meis interactions (Figure 2A). Interaction between PBC and Meis involves the N-terminal PBC-A and Meis-A domains, respectively (Mann and Affolter, 1998). In the absence of Meis, the PBC-A domain is masking two nuclear localization signals located in the HD of PBC. The interaction with Meis relieves the masking activity of the PBC-A domain, allowing the nuclear translocation of PBC (Saleh et al., 2000; Stevens and Mann, 2007).

Interactions between Hox and PBC have been extensively studied at the biochemical and structural levels. All these analyses converge to show a preponderant role for a short conserved motif present in Hox proteins, named hexapeptide (HX) (Mann et al., 2009). The HX motif lies upstream to the HD and contains a core Y/FPWM sequence in all but Abdominal B-group Hox proteins, which have a more divergent sequence (Merabet et al., 2009). More generally, the HX motif is defined as a PBC interaction motif (PIM) that contains an invariant Tryptophan residue located in a hydrophobic environment, followed by basic residues from +2 to +5 (In der Rieden et al., 2003). Crystal structures of vertebrate and invertebrate Hox/PBC complexes solved with anterior, central or posterior Hox proteins point to the critical role of the Tryptophan residue in maintaining strong interactions within the hydrophobic pocket formed in part by the three extra residues of the PBC HD (Passner et al., 1999; Piper et al., 1999; LaRonde-LeBlanc and Wolberger, 2003; Joshi et al., 2007). A recent structural analysis of the Hox/PBC complex bound on a physiological DNA-binding site further underlined that Hox paralog specific residues located in the N-terminal arm of the HD and in the linker region connecting the HX motif to the HD are important for recognizing a specific shape of the DNA minor groove in the presence of PBC (Joshi et al., 2007). SELEX-seq based approaches confirmed that *Drosophila* Hox/PBC complexes preferentially recognize different nucleotide sequences characterized by distinct minor groove topographies (Slattery et al., 2011). These results open new avenues for apprehending the molecular mechanisms underlying Hox and Hox/PBC DNA-binding specificity (Abe et al., 2015). Nevertheless, the systematic involvement of a unique Hox protein motif in the interaction with PBC does not easily explain the broad variety of functions that Hox/TALE complexes have *in vivo* (Hueber and Lohmann, 2008; Mann et al., 2009).

### Specific PBC interactions motifs (SPIMs) as versatile complements to diversify Hox/TALE interaction properties in Bilateria and Cnidaria

Our knowledge of Hox-TALE interaction properties results mostly from *in vitro* approaches. Along the same line, the duplication of Pbx and Meis genes in vertebrates could provide a supplementary layer of complexity. For example, direct Hox-Meis interactions are described with mouse proteins but their functional significance remains to be elucidated (Shen et al., 1997; Williams et al., 2005). The existence of alternative modes in Hox-PBC interaction came from the observation that the HX mutation does not obligatorily affect PBC-dependent functions of Hox proteins in the *Drosophila* embryo (Galant et al., 2002; Merabet et al., 2003). Additionally, several central and posterior Hox proteins from vertebrates and invertebrates interact with the TALE cofactors independently of the HX motif *in vitro* and *in vivo* (Hudry et al., 2012). Interestingly, HX-independent interactions between Hox and PBC are most often observed in the presence of Meis, and the involvement of Meis in such HX-independent interactions actually depends on its DNA-binding near the Hox/PBC binding site (Hudry et al., 2012). In other words, in acting at the level of target cis-regulatory sequences, Meis contributes to diversify the mode of Hox-PBC interactions and thus Hox functions (Merabet and Hudry, 2013).

The flexibility of Hox-TALE interaction properties is predicted to rely on Hox protein motifs that are more gene-specific than the generic HX motif. These motifs are named SPIMs [Specific PBC Interaction Motifs, see also Merabet and Hudry, 2013]. Like the HX motif, SPIMs belong to the so-called short linear motifs, which are classically 5–10 residues long and most often located within intrinsically disordered protein regions (Tompa et al., 2014). Two such motifs have been identified in the *Drosophila* Ultrabithorax (Ubx) and AbdominalA (AbdA) proteins (Merabet et al., 2007, 2011; Hudry et al., 2012). One of them is conserved in insect AbdA proteins, with a core TDWM sequence reminiscent of the HX motif. The other motif, named UbdA, is conserved between the protostome Ubx and AbdA proteins (Balavoine et al., 2002). Recent structural analyses showed that the UbdA motif constitutes a flexible extension of the HD that can establish direct contacts with the PBC partner (Foos et al., 2015). Altogether, studies with Ubx and AbdA confirm that Hox-TALE interactions and functions can rely on species- and/or paralog-specific motifs.

SPIMs remain to be identified in the majority of Hox proteins exerting HX-independent interactions with the TALE cofactors. Still, the usage of different SPIMs in Hox proteins constitutes an appealing molecular strategy for supporting the specific patterning functions of Hox/TALE complexes during development (Figure 2B). Moreover, the conservation of this property in vertebrate and invertebrate species (Hudry et al., 2012) strongly suggests that interaction flexibility is ancient in Bilateria. As a consequence, it is of upmost interest to trace its origin beyond Bilateria and assess its role in developmental and/or patterning functions.

**Figure 3:**
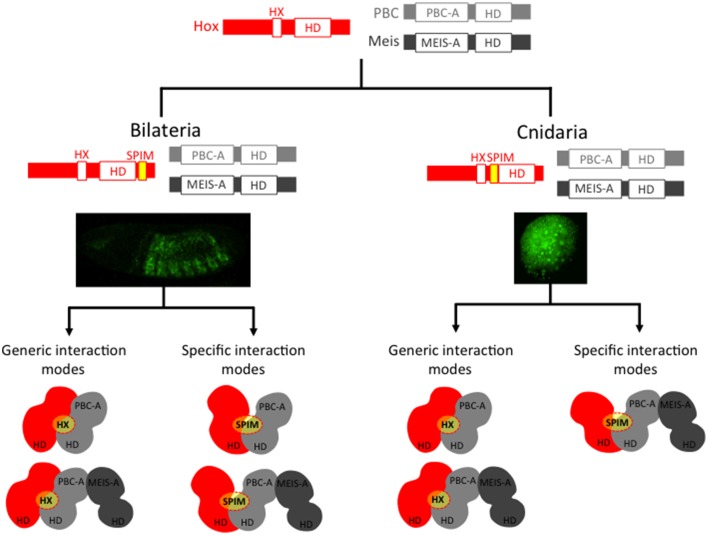
**Cnidarian and bilaterian Hox/TALE networks display similar interaction properties**. Ancestral Hox/TALE networks were strictly relying on the HX motif. The apparition of SPIMs in bilaterian and cnidarian lineages allowed Hox proteins to diversify their interaction modes with the TALE partners. Pictures depict *in vivo* interaction between Hox and PBC proteins in a live *Drosophila* (right) or *Nematostella* embryo, as described in Hudry et al. (2011, 2014). Compared to *Drosophila*, the usage of SPIMs in *Nematostella* Hox proteins is strictly dependent on the presence of Meis (Hudry et al., 2014). The absence of identical SPIMs between bilaterian and cnidarian Hox proteins suggests that these motifs emerged independently in these two groups (see also Figure 4).

Besides Bilateria, Cnidaria is the only other phylum that contains a bona fide Hox repertoire (Chourrout et al., 2006; Kamm et al., 2006). As mentioned previously, the role of cnidarian Hox genes in axis patterning is unclear. Furthermore, not all cnidarian Hox proteins contain an intact HX motif [(Hudry et al., 2014) and Table 1]. Nevertheless, as cnidarians express PBC and Meis genes (Matus et al., 2006; Hudry et al., 2014), a Hox/PBC/Meis network could potentially exist. The interaction properties of Hox, PBC and Meis proteins of the sea anemone *Nematostella vectensis* were recently tested, and as expected, these proteins form dimeric and trimeric complexes *in vitro* (Hudry et al., 2014). In addition, mutating the HX motif leads to the loss of the cnidarian Hox/PBC complex, but this loss is rescued in the presence of Meis. Hence, as observed in bilaterians, the *Nematostella* Meis allows *Nematostella* Hox proteins to use alternative modes of interaction with PBC. Thus, bilaterian and cnidarian Hox proteins share the property of using different interfaces for recruiting the TALE cofactors. We propose that these additional interfaces could correspond to SPIMs that remain to be identified in several instances (Figure 3). Moreover, with the exception of the HX motif, bilaterian and cnidarian Hox proteins do not share strong sequence similarities outside the HD, suggesting that those putative SPIMs could have evolved independently during eumetazoan evolution (see also below).

**Table 1:**
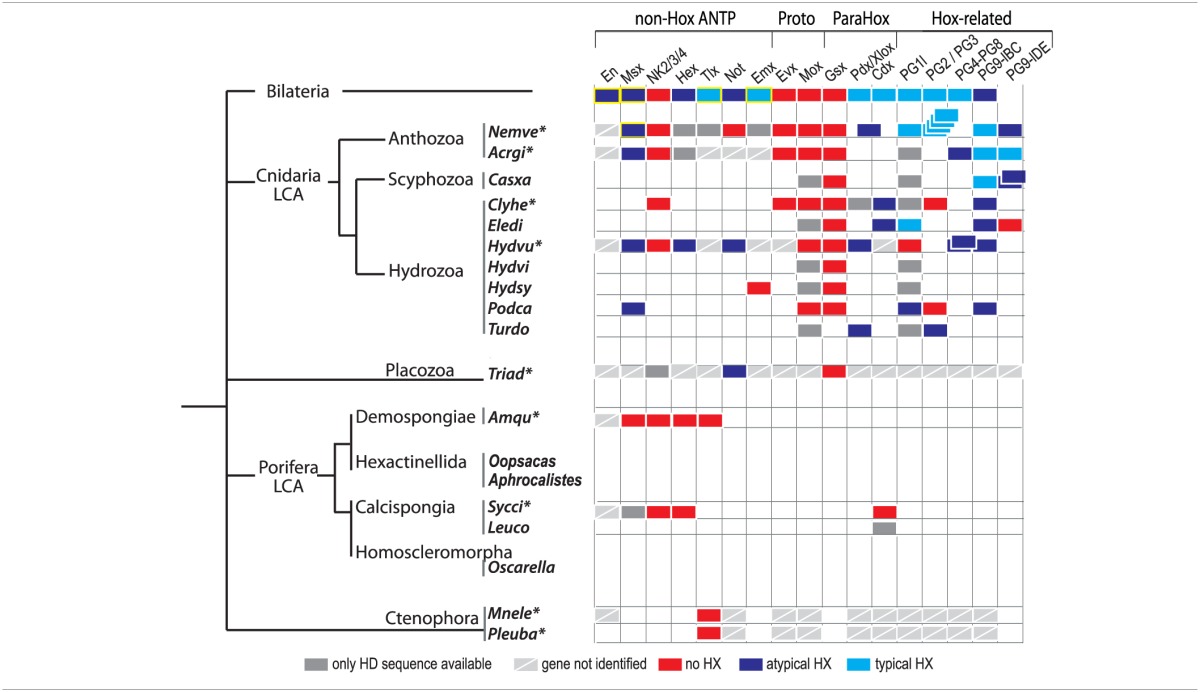
**Presence or absence of the HX motif among the Hox/ParaHox and non-Hox/ParaHox families across Metazoa**.

*The color code denotes for presence or absence of the HX motif, and for incomplete or non-annotated gene, as indicated. Boxes surrounded in yellow in non-Hox/ParaHox proteins highlight a demonstrated role of the HX motif for interaction with TALE partners. See main text for details. Protein sequences were retrieved from Uniprot/Swissprot. Stars denote species with sequenced genome*.

### Genesis of ANTP-TALE networks during early metazoan evolution

Molecular analyses underline that the HX motif is a generic interaction platform for recruiting the TALE partners. We therefore analyzed a large number of available protein sequences for assessing the presence of a putative HX motif in ANTP class members. A peptide sequence was considered as a putative HX motif when containing the consensus Y/FPWM (typical HX motif) or a single W (atypical/divergent HX motif) residue followed by a basic residue (R or K) from +2 to +6 and not localized more than 30 residues away from the HD (Table 1). In Bilateria, the HX motif is found in almost all Hox/ParaHox members, and in several individual representatives of non-Hox/ParaHox protein families, including Engrailed (En), Msx, Hex, Tlx, Not, and Emx proteins (Table 1). The HX motif is found in cnidarian Hox/ParaHox members among early-branched animal phyla. It is however less conserved when compared to Bilateria, being lost or divergent in several cnidarian lineages (Table 1). Atypical HX motifs are also found in Msx and Hex members of Cnidaria, and in Not members of Cnidaria and Placozoa (Table 1). Interestingly, the Evx, Mox, and Gsx proteins, which likely represent the most ancestral ProtoHox and Hox/ParaHox family members (Minguillón and Garcia-Fernàndez, 2003; Quiquand et al., 2009) all lack the HX motif (Table 1).

PBC-recruiting functions have been assigned to few non-Hox proteins among the ANTP class so far. Among them are the mammalian Tlx, *Drosophila* En and *Nematostella* Msx proteins, which do interact in a fully HX-dependent manner with the TALE cofactors (Rhee et al., 2004; Brendolan et al., 2005; Fujioka et al., 2012; Hudry et al., 2014). Still, these proteins display subtle differences in their TALE interaction properties. For example, the *Drosophila* En protein interacts with PBC or PBC/Meis in a HX-dependent manner (Hudry et al., 2014). By comparison, the Msx protein from *Nematostella* interacts in a HX-dependent manner with PBC, but only in the presence of Meis (Hudry et al., 2014). These observations highlight that the role of the PBC/Meis partnership in HX-dependent interactions can be different depending on the protein family and animal lineage considered.

**Figure 4:**
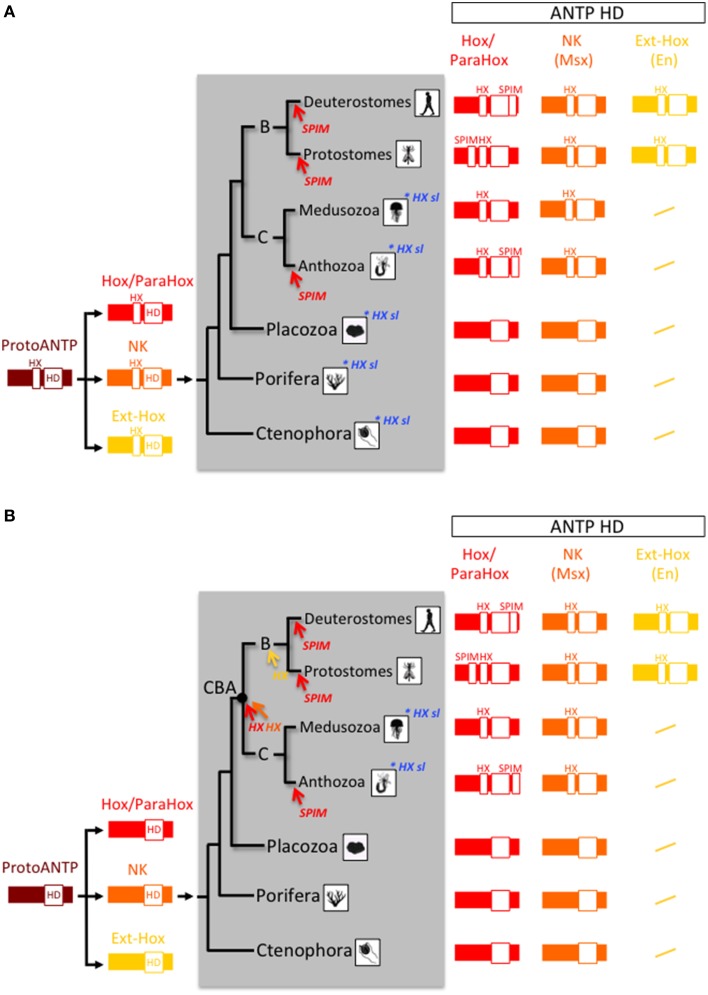
**Two evolutionary scenarios for the origin and early evolution of Hox-TALE interaction properties. (A)** In the first scenario, the HX motif arose in the ProtoANTP gene of the metazoan LCA. There were multiple secondary losses in the Hox/ParaHox (sl; highlighted in blue) and other families (not indicated) in ctenophores, porifers, placozoans and cnidarians. **(B)** In the second scenario, the HX motif appeared independently several times during evolution, acquired in the Hox/ParaHox (red arrow), and NK (Msx, orange arrow) families of the last common ancestor of Cnidaria and Bilateria (CBA), or in Ext-Hox members (as exemplified with En, yellow arrow) of the bilaterian ancestor. The absence of the HX motif in Hox/ParaHox members of several cnidarian species indicates secondary lost events (highlighted in blue; see also Table 1). In both scenarios, the HX motif served as a molecular template for diversifying TALE interaction properties only in the Hox/ParaHox family. This was achieved by the emergence of SPIMs. These motifs were independently acquired (highlighted in red) in Bilateria and Cnidaria, coinciding with strong morphological radiation in these two phyla.

We propose two different evolutionary scenarios to explain the presence of the HX motif in several ANTP family members among metazoan lineages: (i) either the HX motif was already present in the ProtoANTP ancestor, constituting the first molecular interface for recruiting the TALE cofactors (Figure 4A), or (ii) it emerged multiple times independently in the different ANTP families across animal evolution (Figure 4B). The position of the HX motif systematically located in the upstream vicinity of the HD supports the first scenario. As a corollary, the absence of any HX-like motif in all but one (Not) ANTP members of Placozoa, Porifera and Ctenophora would be attributed to repeated secondary losses. Although more sequences are needed in these three early-branched animal phyla, this apparently global and systematic loss of HX motif sequences is intriguing. This could argue in favor of the second scenario, whereby the HX motif would have appeared sporadically by convergent evolution in the different protein families. This second scenario does not exclude additional secondary losses, as observed in cnidarian Hox/ParaHox proteins (Table 1). Moreover, evolution by convergence is not atypical for short motifs in general (Van Roey et al., 2013), and has for example already been proposed for another motif widely found in ANTP-class members (including Gsx, En, Emx, and several NK) and other non-homeoproteins (Williams and Holland, 2000). In the case of the HX motif, it seemingly appeared later during evolution in bilaterian Tlx, Emx and En proteins (Table 1), suggesting a mechanism of convergent evolution. Of note, these bilaterian proteins are known to interact and/or participate with TALE cofactors in the context of tissue-specific functions (Brendolan et al., 2005; Capellini et al., 2010). Along the same line, an HX motif is also present in non-ANTP class proteins, including LIM and several myogenic bHLH proteins (see In der Rieden et al., 2003, for a more complete list of HX-containing proteins). In the case of bHLH proteins the HX motif was further shown to be involved in the interaction and function with TALE cofactors during skeletal muscle differentiation in vertebrates (Knoepfler et al., 1999; Maves et al., 2007, 2009; Yao et al., 2013). Together these observations highlight the strong evolutionary plasticity of the HX motif for providing a TALE-recruiting activity to highly divergent protein families.

### Genesis of the Hox-TALE patterning system during metazoan evolution

The evolutionarily conserved PBC-A and Meis-A domains in PBC and Meis proteins are restricted to Bilateria, Cnidaria and Placozoa, suggesting that a Hox/TALE network exists only in these three phyla (Figure 1). Like all cnidarian and bilaterian Gsx proteins, the unique ParaHox Gsx representative of *Trichoplax adhaerens* has no HX motif (Table 1) and cannot interact with PBC and Meis (Hudry et al., 2014). By contrast, the two other ParaHox and the Hox-related proteins have retained an HX motif in most cnidarians and bilaterians (Table 1). Thus, Cnidaria and Bilateria are the only phyla where a Hox-TALE interaction network is effective.

Since interaction with TALE proteins is not a specific feature of Hox proteins, the next question is “When did Hox proteins acquire their patterning functions linked to the interaction with the TALE cofactors?” We postulate here that the acquisition of differential patterning functions was tightly linked to the emergence of diversified interaction properties between Hox and TALE proteins. Then the question could be reformulated as: “When did alternative TALE interaction motifs appear in addition to the HX motif in the Hox/ParaHox family?”

**Figure 5:**
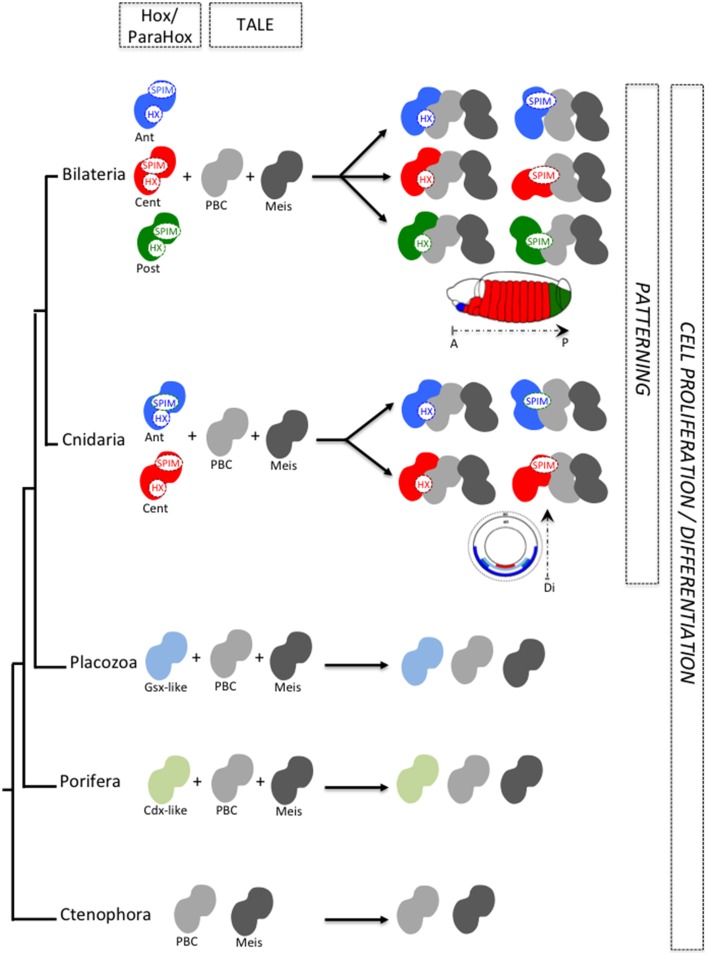
**SPIMs as molecular markers of a Hox/TALE patterning system during animal evolution**. The acquisition of SPIMs in Cnidaria (*Nematostella*) and Bilateria (*Drosophila*, mammals) allowed Hox proteins to diversify their interaction modes with the TALE partners. This molecular diversification was essential for providing differential activities to Hox proteins along the longitudinal axis. Illustrative examples are provided along the anterior-posterior axis of the *Drosophila* embryo or along the directive axis of the *Nematostella* embryo. Anterior (ant), central (cent), and posterior (post) Hox proteins are depicted by a different color. In Placozoa and Porifera, ParaHox-like members are present but these proteins do not contain any HX motif. Along the same line, placozoan and poriferan PBC and Meis representatives lack the PBC-A and MEIS-A domains (see Figure 1) and thus cannot interact together. As a consequence, TALE interaction networks do likely not exist in those two phyla. We postulate that Hox/ParaHox transcription factors were initially dedicated to cell proliferation/cell differentiation with no patterning function, whereas Hox/TALE interactions co-evolved with patterning functions.

Recent work with *Nematostella* Hox and TALE proteins (Hudry et al., 2014) suggests that SPIMs co-evolved with the specification of embryonic axes. As SPIMs are specific to a given Hox family or to a given species, they likely emerged independently several times during evolution (Figure 4). We propose that the original HX-dependent interaction mode served as an initial molecular template for experiencing these novel HX-independent interaction properties with the TALE partners. It is tempting to speculate that SPIMs were a molecular prerequisite for allowing Hox proteins to acquire patterning functions during early eumetazoan evolution. In this model, the acquisition of SPIMs in Hox proteins likely happened in parallel to mechanisms regulating their expression, allocating Hox genes to specific spatio-temporal domains along the longitudinal axis (Figure 5).

Finally, SPIMs do not necessarily correspond to related peptide sequences, as already noticed for the TDWM and UbdA motifs in *Drosophila* (Merabet and Hudry, 2013), making their identification difficult. Additional SPIMs need however to be identified to validate our model. Several tools are now available for predicting the presence of short interaction motifs in protein sequences, based on the analysis of amino acid chemical properties and the classification of hundreds of characterized short motifs in databases (Tompa et al., 2014). Interestingly, these tools predict a number of short motifs in several regions of bilaterian (Merabet and Dard, 2014) and cnidarian (Baëza et al., 2015) Hox proteins. These regions are often involved in the interaction with different TFs (Baëza et al., 2015), and could therefore contain good candidate SPIMs to test in the future.

### Perspective: the HX motif and SPIMs as molecular markers of patterning functions in the ParaHox family?

ParaHox genes share several common features with the Hox genes. For example, they are organized in clusters and display spatial-temporal constraints for their expression during embryogenesis of several bilaterian species (Garstang and Ferrier, 2013). The expression profile of ParaHox genes in Cnidaria is also reminiscent of important functions during embryogenesis, regeneration or budding, as seen in the solitary polyp *Hydra* (Schummer et al., 1992; Miljkovic-Licina et al., 2007), the coral *Acropora* (Hayward et al., 2001), the jellyfish Podocoryne (Yanze et al., 2001), the sea anemone Nematostella (Finnerty et al., 2003), or the colonial polyp *Hydractinia* (Cartwright et al., 2006). Moreover, ParaHox genes, and more particularly Gsx, could be more representative of the ProtoHox ancestor gene than any other Hox gene (Quiquand et al., 2009). Although Gsx does not contain any HX motif, it has a conserved role for the specification of neuroblast lineages in bilaterians (Weiss et al., 1998; Waclaw et al., 2009; Winterbottom et al., 2010; López-Juárez et al., 2013) and cnidarians, with a fine regulation along the body axis (Hayward et al., 2001; Miljkovic-Licina et al., 2007). Along the same line, Pdx-1 plays a crucial role in pancreatic beta-cell differentiation (Kaneto et al., 2007). These observations suggest that a primordial ParaHox (and Hox) function was dedicated to the emergence of novel cell types along the body axis, possibly in a TALE-independent manner (Figure 5) (De Jong et al., 2006; Miljkovic-Licina et al., 2007; Quiquand et al., 2009). This role could then have been deployed in several Hox/ParaHox members and in different tissues, requiring the acquisition of additional molecular features such as the HX motif and SLIMs for diversifying the novel patterning functions. In agreement with this hypothesis, in Bilateria Pdx/Xlox and Cdx transcription factors are required for the patterning of endodermal derivatives (Cole et al., 2009; Beck and Stringer, 2010; Annunziata et al., 2013; Ikuta et al., 2013) or during axis elongation with the Hox genes (Moreno and Morata, 1999; Van den Akker et al., 2002; Shinmyo et al., 2005; Young et al., 2009). The impact of TALE cofactors in those patterning functions remains to be investigated. The role of Pdx/Xlox and Cdx is also largely unknown in cnidarians. Testing their interaction properties with TALE cofactors could undoubtedly provide new insightful information into the origin and evolvability of the Hox/TALE patterning system in Metazoa. Ultimately, such studies should tell us whether the combination of one HX motif plus several SPIMs in the ParaHox proteins was necessary and sufficient to promote a spatial organization of cell differentiation along the body axis and thus the emergence of patterning functions in different tissues.

## Conclusion

Hox proteins are TFs displaying highly similar DNA binding properties *in vitro*. Still, each Hox protein will dictate a specific developmental program with the same set of TALE cofactors. We proposed here that the apparition of a functional Hox/TALE patterning system during metazoan evolution was tightly linked to the acquisition of different short motifs named SPIMs. The usage of different SPIMs in Hox proteins constitutes an appealing molecular strategy for explaining the specific and various developmental functions of Hox/TALE complexes. Due to their small size, SPIMs present the advantage of being highly dynamic during evolution, allowing diversifying the molecular code between Hox and TALE proteins. This model supposes that interaction flexibility is an important feature of the Hox/TALE patterning system. Whether this molecular strategy could more widely apply to other key patterning networks constitutes a major issue to investigate in the future.

### Conflict of interest statement

The authors declare that the research was conducted in the absence of any commercial or financial relationships that could be construed as a potential conflict of interest.

## Abbreviations

ANTP: Antennapedia
AP: anterior posterior
HD: Homeodomain
HX: Hexapeptide
PG: Paralog Group
SPIM: Specific PBC Interaction Motif
TALE: Three Amino acid Loop Extension
TF: Transcription factor.
